# A fluorescence polarization assay for high-throughput screening of inhibitors against HIV-1 Nef-mediated CD4 downregulation

**DOI:** 10.1016/j.jbc.2024.107528

**Published:** 2024-07-02

**Authors:** Mohammad Karimian Shamsabadi, Xiaofei Jia

**Affiliations:** 1 Department of Chemistry and Biochemistry, University of Massachusetts Dartmouth, Dartmouth, Massachusetts, USA; 2 The Biomedical Engineering and Biotechnology Program, University of Massachusetts Dartmouth, Dartmouth, Massachusetts, USA

**Keywords:** HIV, Nef, CD4, antiretroviral, high-throughput screening, fluorescence polarization

## Abstract

Therapeutic inhibition of the viral protein Nef is an intriguing direction of antiretroviral drug discovery—it may revitalize immune mechanisms to target, and potentially clear, HIV-1–infected cells. Of the many cellular functions of Nef, the most conserved is the downregulation of surface CD4, which takes place through Nef hijacking the clathrin adaptor protein complex 2 (AP2)-dependent endocytosis. Our recent crystal structure has unraveled the molecular details of the CD4–Nef–AP2 interaction. Guided by the new structural knowledge, we have developed a fluorescence polarization–based assay for inhibitor screening against Nef’s activity on CD4. In our assay, AP2 is included along with Nef to facilitate the proper formation of the CD4-binding pocket and a fluorescently labeled CD4 cytoplasmic tail binds competently to the Nef–AP2 complex generating the desired polarization signal. The optimized assay has a good signal-to-noise ratio, excellent tolerance of dimethylsulfoxide and detergent, and the ability to detect competitive binding at the targeted Nef pocket, making it suitable for high-throughput screening.

AIDS, caused by HIV infections, remains one of the deadliest diseases affecting human lives. As estimated by the World Health Organization, about 38.4 million people were living with HIV infections at the end of 2021, and a total of 650,000 people died from AIDS or other HIV-related causes in the year of 2021. Antiretroviral therapy (ART) has dramatically reduced the death rate, and thus transformed the treatment, of HIV infections. Current antiretrovirals used in ART target either viral enzymes (protease, integrase, or reverse transcriptase) or viral entry. Although they can effectively block viral transformation, they are not curative; latent viral reservoirs persist in the infected individuals, and, if ART is stopped, viral rebound occurs within weeks (1). Due to such limitations, treatment of HIV-1 infections needs to be continuous and lifelong. However, such prolonged treatment often results in drug resistance and/or severe side effects such as metabolic disorders and cardiovascular complications (2). Novel antiretrovirals that can better treat, or even eliminate, HIV infections are therefore highly desired.

An attractive direction of developing novel antiretrovirals is the therapeutic inhibition of the viral accessory protein Nef. Through downregulating cellular immune factors, Nef enables the infected cell to evade host immunity and thus plays an important role in viral pathogenesis. Defects in *nef* genes are associated with disease nonprogression—patients infected by such HIV-1 strains do not develop AIDS for decades in the absence of ART (3, 4, 5, 6). The promise of Nef inhibition is that it may revitalize immune surveillance mechanisms leading to killing of infected cells by the empowered immune cells. This may be particularly relevant in the proposed shock and kill strategy (7, 8, 9): following latency reversal, clearance of replication-competent cells by host immunity may benefit significantly from Nef inhibition.

Among the many cellular functions of Nef, CD4 downregulation is the most conserved (10). Although CD4 is the primary receptor for HIV infections, its cellular presence post-entry negatively impacts viral replication and transmission. Association between CD4 and the viral Env protein would interfere with the processing and anterograde transport of Env as well as impede new virion release at the cell surface (11, 12, 13, 14). Furthermore, surface CD4 would cause superinfection, leading to premature cell death and thus limiting viral replication (15, 16). Finally, binding between CD4 and Env at the cell surface would expose antigenic Env epitopes, sensitizing the infected cell to antibody-dependent cellular cytotoxicity (17, 18).

Nef downregulates surface CD4 *via* hijacking the clathrin AP2–dependent endocytosis, which subsequently leads to degradation of CD4 in the lysosomes (19, 20, 21). A recent crystal structure solved by us has revealed the molecular details of the CD4-Nef-AP2 association (22). As shown by the structure, Nef uses a conserved pocket to recruit the CD4 cytoplasmic domain (CD4_CD_), and the overall shape of the CD4-binding pocket is dependent on the Nef-AP2 association (Fig. 1*A*). Intriguingly, it has been shown, by another structure of ours, that the same Nef pocket is used for binding and downregulating major histocompatibility complex class I (MHC-I), although the binding site there is shaped differently through the association between Nef and the hijacked clathrin AP1 complex (23). These structural findings strongly suggest that this common pocket on Nef should be the focus of inhibitor screening; they also indicate that the specific cellular factor hijacked by Nef should be incorporated in the assay when screening for inhibitors against the corresponding function of Nef. Furthermore, the CD4-binding pocket, which is constructed partially by Nef’s N-terminal flexible arm (Fig. 1*A*), is of intermediate size: the interface between CD4 and Nef covers an area of only 887 Å^2^. This suggests that the pocket should be susceptible to inhibition by small molecules. Inspired and guided by these structure-derived insights, we have designed, developed, and optimized a fluorescence polarization (FP) assay for screening inhibitors against Nef-mediated CD4 downregulation.

**Figure 1:**
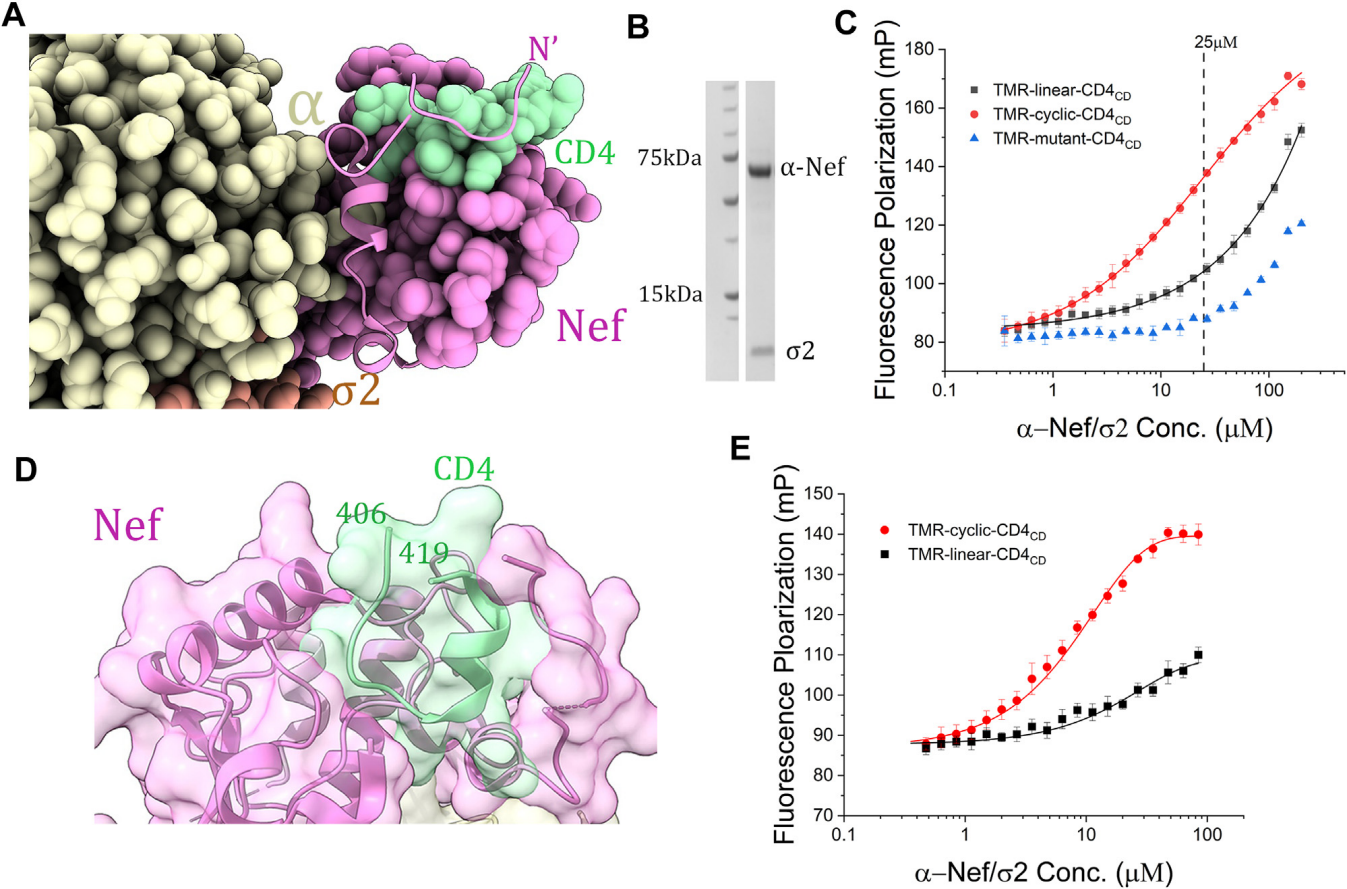
**Structure-guided design and initial tests of the FP assay.***A*, the N terminus of Nef (*cartoon*) becomes ordered upon AP2 association and participates in CD4 binding (PDB ID: 6URI). *B*, SDS-PAGE of purified α-Nef/σ2. *C*, Nef-bound CD4_CD_ (406–419) adopts a near-circular shape. *D*, FP assays assessing the binding between the fluorescent probes and α-Nef/σ2. The TMR-mutant-CD4_CD_ curve represents both the baseline (at lower concentrations of α-Nef/σ2) and the interference caused by light scattering of α-Nef/σ2 at higher concentrations. FP signals from using TMR-linear-CD4_CD_ as the probe are above the baseline but modestly so. FP signals from using TMR-cyclic-CD4_CD_ as the probe are sufficiently higher than the baseline. *E*, FP signals after subtraction of the background noise. AP2, adaptor protein complex 2; CD4_CD_, CD4 cytoplasmic domain; FP, fluorescence polarization; TMR, tetramethylrhodamine.

## Results

### Construct design and initial FP assay using TMR-linear-CD4_CD_

We followed the three-step protocol described by Moerke to develop an *in vitro* FP assay for high-throughput screening (HTS) of inhibitors against Nef-mediated CD4 downregulation (24). We first designed and commercially synthesized the fluorescent probe: a linear CD4_CD_ peptide with a fluorescence tag, tetramethylrhodamine (TMR), attached at the N terminus. We then engineered a Nef-AP2 construct competent in binding CD4_CD_ and suitable for the FP assay. As shown by our recent structure (22), CD4 binding is facilitated by the Nef-AP2 association—the otherwise flexible N-terminal loop of Nef becomes well-ordered upon binding to AP2 and a portion of this loop then engages productively in binding CD4 (Fig. 1*A*). This suggests to us that the binding (and the FP assay) would be the most competent if Nef and AP2 are stably bound. There are two challenges in maintaining a stable complex of Nef and AP2 in solution. First, the tetrameric AP2 complex tends to stay in the closed conformation, which is incapable of binding Nef. This could be dealt with by using a truncated version of AP2 containing the N-terminal two-thirds (residues 1–398) of the α subunit and the σ2 subunit. Such a hemicomplex has its substrate-binding site fully exposed and has been shown to bind Nef *in vitro* (25). Furthermore, according to our structure, Nef, when bound to this hemicomplex of AP2, should be fully competent in recruiting CD4_CD_ (22). The second challenge is that binding between the α/σ2 hemicomplex and Nef is still of moderate affinity (25). To overcome this challenge and ensure a stable association between Nef and α/σ2, we created a fusion protein by fusing Nef to the C terminus of α (1–398) *via* a 31-aa linker, which should be flexible enough to not interfere with Nef’s binding with α/σ2. The α-Nef fusion was coexpressed with σ2, and the complex of α-Nef/σ2 was conveniently purified to homogeneity in high yield (Fig. 1*B*).

The above constructs—TMR-linear-CD4_CD_ and the α-Nef/σ2 protein—indeed gave the expected signal in the FP assay (Fig. 1*C*). Here, we selected 200 nM as the working concentration of TMR-linear-CD4_CD_ for the FP assay because, at this concentration of the probe, intensity of the emitted total fluorescence is more than 10-fold higher than that of the background (buffer only).

Importantly, we found that a significant amount of noise exists in the observed FP signal. Such noise is best revealed by the usage of a TMR-mutant-CD4_CD_, which carries three mutations—I410D, L413D, and L414D—that completely abolish CD4’s ability to bind Nef (22). FP assay using this TMR-mutant-CD4_CD_ showed that, at concentrations of α-Nef/σ2 higher than 60 μM, FP signal above the baseline was observed despite that TMR-mutant-CD4_CD_ is incapable of binding (Fig. 1*C*). We believe that such noise should have come from light scattering by high concentrations of α-Nef/σ2 (26). Indeed, the α-Nef/σ2 protein, in the absence of the fluorescent probe, produced a positive FP signal in a concentration-dependent manner (data not shown).

While the undesired light scattering demands that we use low concentrations of α-Nef/σ2 in the FP assay, the low binding affinity between α-Nef/σ2 and TMR-linear-CD4_CD_ dictates, however, that binding only happens at high concentrations of α-Nef/σ2. As a result, the range of workable α-Nef/σ2 concentrations is very narrow, and within it the FP signal window is at best modest (Fig. 1*C*).

### Using TMR-cyclic-CD4_CD_ improves the competency of the FP assay

We then reasoned that, to improve the FP assay, we need a fluorescent probe that is capable of binding α-Nef/σ2 with higher affinity. The structure, once again, helped us design such a probe. As shown by the structure, CD4_CD_ adopts a near-circular shape when bound in the hydrophobic pocket of Nef (Fig. 1*D*). We therefore hypothesized that a cyclized CD4_CD_ peptide should bind competently to α-Nef/σ2 and should do so more efficiently than the linear CD4_CD_, because binding with the former should be more favorable entropically. We thus designed and commercially synthesized a TMR-cyclic-CD4_CD_ probe. When tested in the FP assay, the TMR-cyclic-CD4_CD_ showed binding at lower concentrations of α-Nef/σ2, than that of TMR-linear-CD4_CD_, indicating higher affinity binding was indeed achieved through this design (Fig. 1*C*). After subtracting out the noise signal (see details in Experimental section), a K_D_ of 9.8 μM was derived for the binding between the TMR-cyclic-CD4_CD_ probe and the α-Nef/σ2 protein, while the K_D_ for the TMR-linear-CD4_CD_ probe could not be accurately derived because the binding failed to reach saturation despite of the high concentrations of α-Nef/σ2 used (Fig. 1*E*). Importantly, the usage of TMR-cyclic-CD4_CD_ significantly increases the signal window of the assay. To ensure that the assay is sufficiently sensitive to competition, the α-Nef/σ2 concentration of 25 μM was selected, which gives a signal window of around 50 mP.

### Assay optimization through incorporation of detergents

**Figure 2:**
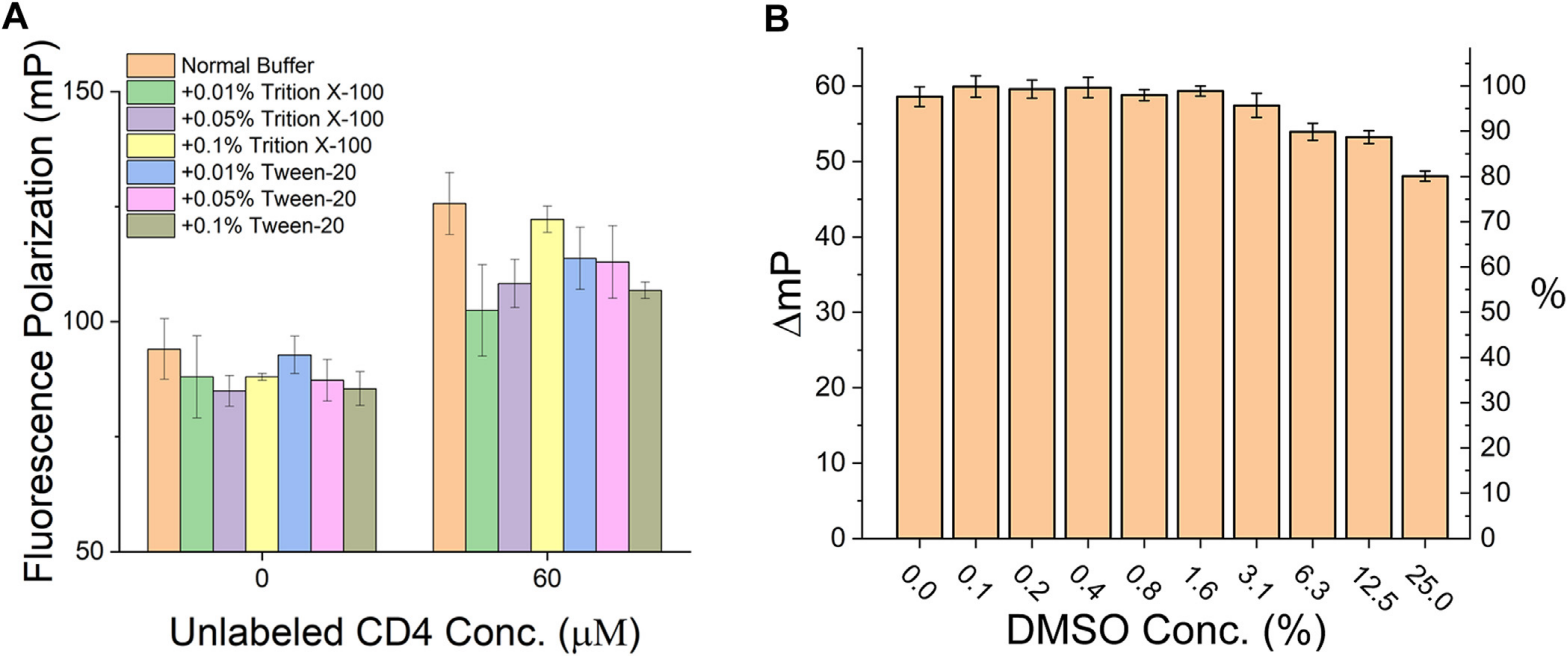
**Effects of detergents and DMSO on the FP assay.***A*, 200 nM TMR-cyclic-CD4_CD_ was either alone in solution or mixed with 60 μM unlabeled CD4_CD_. Buffers contain either no or different concentrations of detergent. FP signals were read after 2-h incubation. While the FP signals of TMR-cyclic-CD4_CD_ alone (the group of columns on the *left*) were slightly decreased by the presence of detergents, more substantial reductions in FP signals were observed in samples containing both TMR-cyclic-CD4_CD_ and 60 μM unlabeled CD4_CD_ (the group of columns on the *right*) with the greatest reduction seen in 0.01% Triton X-100. *B*, the DMSO tolerance of the assay. *Left y-axis:* FP signal (mP) of the assay normalized by substracting out the background (buffer only) FP signal; the obtained ΔmP values here should correlate directly with binding. *Right y-axis:* percentage of the FP signal correlated with binding (100% corresponds to the FP signal of the binding without DMSO added; 0% corresponds to the no-binding background). Analysis using one-way ANOVA indicates that these data are statistically significant (*p* < 0.0001). CD4_CD_, CD4 cytoplasmic domain; DMSO, dimethylsulfoxide; FP, fluorescence polarization; TMR, tetramethylrhodamine.

During our experiments, we noticed that CD4_CD_ is prone to aggregation: adding unlabeled CD4_CD_ to the TMR-cyclic-CD4_CD_ probe led to increased FP signals (Fig. 2*A*), which is most consistent with TMR-cyclic-CD4_CD_ binding nonspecifically (or aggregating) with unlabeled CD4_CD_. This is presumably due to the presence of multiple hydrophobic residues within the short CD4_CD_ peptide. To mitigate this issue and improve the assay, we tried adding detergent—either Tween 20 or Triton X-100—at different concentrations. As shown in Figure 2*A*, among all tested, 0.01% (v/v) Triton X-100 worked the best in reducing (although not eliminating) the aggregation-related signal. We therefore included 0.01% Triton X-100 in the buffer for all subsequent experiments; no adverse effect was observed from it.

### Assessment of the assay’s tolerance toward DMSO

Since compounds in most small molecule libraries are dissolved in DMSO, dimethylsulfoxide (DMSO), we investigated whether, and to what extent, our FP assay is stable in DMSO. As shown in Figure 2*B*, DMSO concentrations of 3% or lower led to minimal, if at all, decrease of the FP signal. At DMSO concentrations of 6.3%, 12.5%, and 25.0%, the FP signal window (ΔmP) decreased by 10.1%, 11.2%, and 19.8%, respectively. Overall, our FP assay is very stable at up to 3% of DMSO and is thus suitable for library screening.

### The optimized assay is responsive to competitive binding of inhibitors

**Figure 3:**
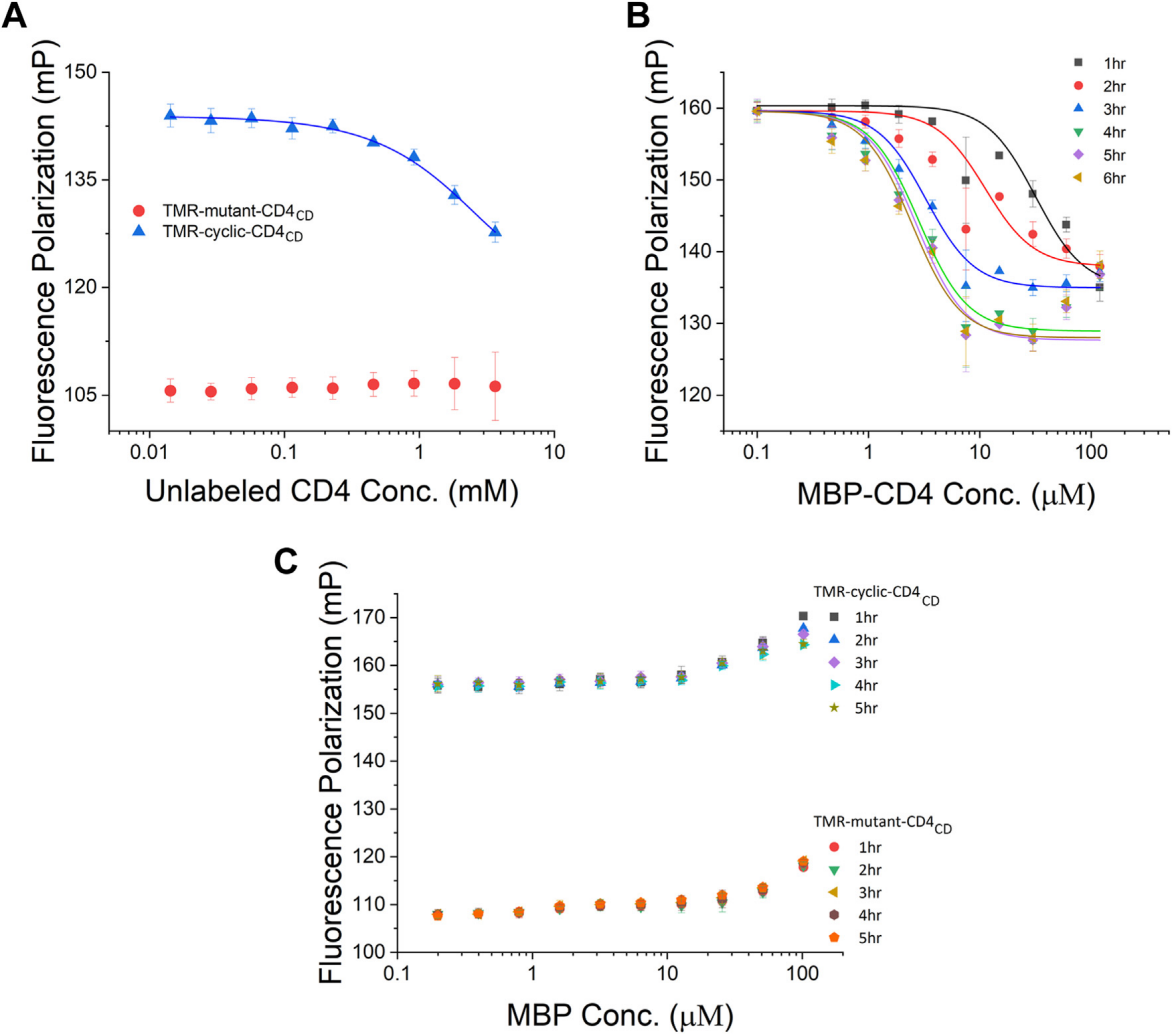
**Dose-dependent competition of the fluorescence-labeled probe by unlabeled CD4**_**CD**_**peptide or MBP-CD4**_**CD**_**.***A*, unlabeled CD4_CD_ showed dose-dependent competition against TMR-cyclic-CD4_CD_ while the control using TMR-mutant-CD4_CD_ showed baseline signals without dose-dependent decrease. *B*, MBP-CD4_CD_ showed dose-dependent competition against TMR-cyclic-CD4_CD_. Incubation up to 4 h improved the competition, but longer incubation did not improve it further. Dose effect was observed up to 30 μM of MBP-CD4_CD_ for all incubation time. For incubation longer than 2 h, when the concentration of MBP-CD4_CD_ increased past 30 μM, the FP signals increased. *C*, in contrast, adding MBP did not result in dose-dependent competition with TMR-cyclic-CD4_CD_ (*top portion*). Similar trend was observed when TMR-mutant-CD4_CD_ was used as the probe (*bottom portion*). In both cases, the FP signal modestly increased at high concentrations of MBP, and the signal was insensitive to incubation. CD4_CD_, CD4 cytoplasmic domain; FP, fluorescence polarization; MBP, maltose-binding protein; TMR, tetramethylrhodamine.

To assess the FP assay’s ability to detect competitive inhibition, we first tested whether unlabeled linear CD4_CD_ can displace TMR-cyclic-CD4_CD_. As shown in Figure 3*A*, addition of unlabeled CD4_CD_ led to a dose-dependent decrease of the FP signal consistent with a competitive binding scenario; in contrast, the control using TMR-mutant-CD4_CD_ as the probe showed stable, baseline polarization signal throughout. These results suggest that the competitor is not damaging the fluorophore in any way and that the observed dose-dependent decrease of the FP signal should be due to competition between the unlabeled linear CD4_CD_ and the TMR-cyclic-CD4_CD_ probe. However, it appears that unlabeled CD4_CD_, even at the highest concentration tested (3 mM), did not displace the bound TMR-cyclic-CD4_CD_ completely (Fig. 3*A*). As mentioned earlier, the short CD4_CD_ peptide contains several hydrophobic residues, and therefore is prone to aggregation. We suspect that some microscopic aggregates of unlabeled CD4_CD_ may be present, and as a result, the monodispersed, competition-competent CD4_CD_ may be just a fraction of the total unlabeled CD4_CD_.

We then tried to make the competitor CD4_CD_ peptide more soluble and less aggregation prone. To do so, we attached a solubility-enhancing maltose-binding protein (MBP) to the N terminus of the CD4_CD_ peptide. When tested in the FP assay, this MBP-CD4_CD_ protein caused dose-dependent decrease of FP at apparent concentrations lower than that of unlabeled CD4_CD_ (Fig. 3*B*); this seems to agree with our hypothesis above that concentrations of competition-competent unlabeled CD4_CD_ may be lower than its apparent concentrations. Interestingly, competition by MBP-CD4_CD_ benefitted from incubation: for the first 4 h of incubation but not afterward, the FP signals progressively decreased (Fig. 3*B*).

As indicated by the TMR-mutant-CD4_CD_ curve (Fig. 3*A*, red curve), when the probe is completely dissociated from α-Nef/σ2, the FP signal should drop to around 105 mP. Yet, in the competition with MBP-CD4_CD_, the FP signal decreased only to ∼127 mP (Fig. 3*B*), which indicates that the displacement of the probe also may not be complete here. In fact, after reaching the lowest FP values at the MBP-CD4_CD_ concentration of around 30 μM, the curves (especially those of 3-h incubation or longer) started to rise as the MBP-CD4_CD_ concentration further increased. We believe that this may be due to either nonspecific binding of TMR-cyclic-CD4_CD_ to MBP-CD4_CD_ aggregates (similar to what was observed between TMR-cyclic-CD4_CD_ and unlabeled CD4_CD_ in Fig. 2*A*) or light scattering by MBP-CD4_CD_, both of which would occur more readily at higher concentrations of MBP-CD4_CD_ leading to increased FP signals.

To further prove the specificity of this assay and further investigate the nature of the noise, we performed another control experiments using MBP (without CD4_CD_ fused to it) as a mock competitor. Under optimized conditions with TMR-cyclic-CD4_CD_ used as the probe, no dose-dependent decrease was observed; instead, at concentrations of MBP higher than 20 μM, the FP signal experienced a gradual, modest increase (top portion of Fig. 3*C*). This indicates that MBP cannot compete with TMR-cyclic-CD4_CD_ for binding the α-Nef/σ2 protein, which further confirms that the dose-dependent decrease of FP signal observed in Figure 3*B* should be due to competition between MBP-CD4_CD_ and the TMR-cyclic-CD4_CD_ probe. Thus, our optimized FP assay is indeed capable of detecting competitive inhibition at the targeted pocket of Nef.

Interestingly, when MBP was used to “compete” against the TMR-mutant-CD4_CD_ probe, which is unable to associate with α-Nef/σ2, the trend observed was similar to that when MBP was used against the TMR-cyclic-CD4_CD_ probe (Fig. 3*C*). Given that TMR-mutant-CD4_CD_ lacks hydrophobic residues and is thus very soluble, we believe that the background noise revealed in this experiment should not come from aggregation of the fluorescent probes but should be caused by light scattering by MBP, a 42-kDa protein (Fig. 3*C*). Given that MBP-CD4_CD_ and MBP are similar in size, it is perhaps reasonable to believe that light scattering by high concentrations of MBP-CD4_CD_ is the major source of noise observed in Figure 3*B*.

Notably, data in Figure 3*C* also confirms that, in the optimized assay, binding between TMR-cyclic-CD4_CD_ and α-Nef/σ2 reaches equilibrium within 1 h of incubation—the FP signal here reached ∼155 mP after 1 h of incubation and remains stable afterward for at least another 4 h. It should also be noted that the superb stability of the signal exhibited here is in great contrast with the observed time effect on the FP signal when MBP-CD4_CD_ was used as a competitor (Fig. 3*B*). We postulate that the time effect observed during competition by MBP-CD4_CD_ was likely caused by a low off-rate (*k*_*off*_) of the TMR-cyclic-CD4_CD_ probe.

Overall, the observed dose-dependent competitions indicate that our optimized FP assay is capable of detecting, and is sensitive to, competitive binding against TMR-cyclic-CD4_CD_. Furthermore, the lack of complete displacement of TMR-cyclic-CD4_CD_ by either unlabeled CD4_CD_ or MBP-CD4_CD_ indicates that, to evaluate the assay’s competency for HTS, neither of these two competitors can serve competently as the positive control.

### The FP assay is robust and suitable for HTS

**Figure 4:**
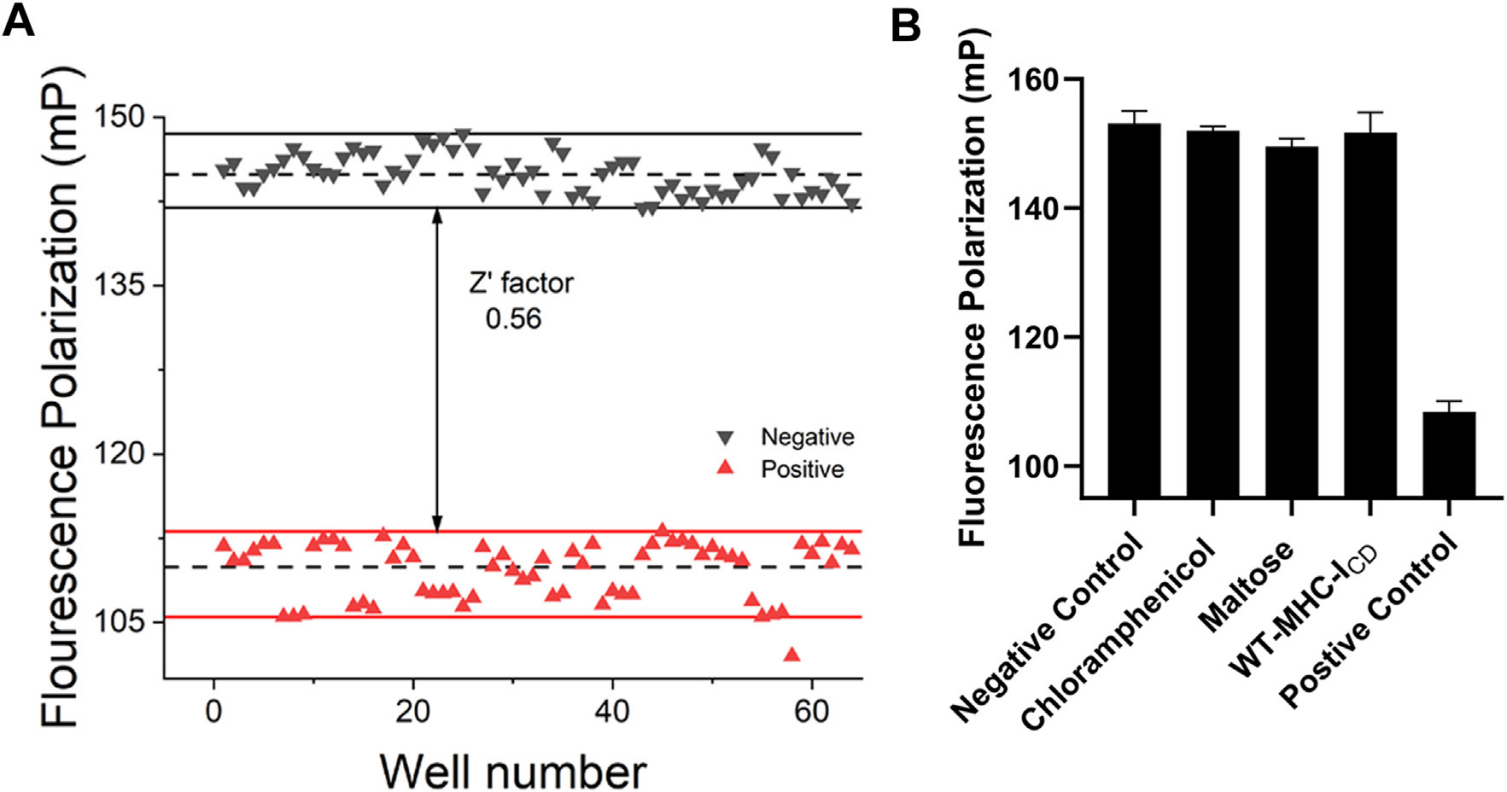
**Assessing the assay’s suitability for HTS.***A*, measurement of the Z′ factor. The mean values of positive and negative controls are indicated by the *dashed lines*, while *solid lines* indicate the range where data points were considered (one data point in the positive controls was considered as an outlier). The Z′ factor was calculated to be 0.56. *B*, tool compounds did not cause noise in the assay. Chloramphenicol, maltose, and MHC-I_CD_ peptide (319–330 of MHC-I) was each dissolved in dimethylsulfoxide and then added to the assay solution to a final concentration of 300 μΜ (final dimethylsulfoxide concentration = 3%). MHC-I, major histocompatibility complex class I.

To assess whether our assay is competent for HTS, we calculated the *Z*′ factor. Given the incomplete competition by either unlabeled CD4_CD_ or MBP-CD4_CD_ (Fig. 3, *A* and *B*), we instead decided to use a solution of TMR-mutant-CD4_CD_ mixed with α-Nef/σ2 as the positive control. Such a control resembles the scenario where the fluorescence probe is completely displaced by an inhibitor and thus should help reveal the true signal window of our assay. From 66 positive controls (α-Nef/σ2 with TMR-mutant-CD4_CD_) and 66 negative controls (α-Nef/σ2 with TMR-cyclic-CD4_CD_), the *Z*′ factor was calculated to be 0.56 (Fig. 4*A*).

Finally, to get a glimpse of whether addition of compounds might or might not lead to significant noise in our assay, we tested the addition of three tool compounds: maltose, chloramphenicol, and MHC-I_CD_ peptide. None of these compounds is expected to inhibit the Nef–CD4 interaction. In particular, while Nef targets the cytoplasmic domain of MHC-I for its downregulation in cells, binding between Nef and MHC-I_CD_ is not expected to occur *in vitro* in the absence of clathrin AP1 or its μ1 subunit (23). As shown in Figure 4*B*, consistent with their lack of activity, addition of these compounds at 300 μΜ did not decrease the FP signal in any meaningful way. Importantly, no significant noise was observed in any case. Overall, the measured *Z′* factor and the validation with tool compounds together indicate that our assay is competent and suitable for HTS (24).

## Discussion

We have developed a robust FP-based assay that is suitable for HTS of small molecule inhibitors against HIV-1 Nef-mediated CD4 downregulation. Our work here benefitted greatly from the high-resolution structure that we solved earlier (22); it not only guided our protein engineering efforts aimed at stabilizing Nef into the desired conformation but also inspired us to use a cyclized version of the CD4_CD_ peptide in our probe design, which helped us gain the needed signal window. Thus, our work here underscores the key role of high-resolution structures in enabling and facilitating drug discovery efforts against challenging targets.

One issue we had in developing this FP assay is the noise signal, which was typically observed when the protein concentration used was high. With a control experiment using MBP as a mock competitor and a soluble, binding-incompetent TMR-mutant-CD4_CD_ as the probe (Fig. 3*C*), we showed that the noise observed in our competition should be mainly due to light scattering by the sizable MBP or MBP-CD4_CD_. Importantly, since library compounds are small and therefore unlikely to cause significant light scattering, such noise may not be present during HTS. Of course, some small molecule may form aggregates, which may lead to light scattering and thus interfere with HTS; such an issue, if indeed occurs, might be mitigated by the presence of 0.01% Triton X-100 in our assay solution.

Parallel to this work, we have also developed, as reported in the companion publication (27), a similar FP assay for inhibitor screening against HIV-1 Nef-mediated MHC-I downregulation. The two FP assays share similar time windows (∼4 h) and comparable Z′ factors. Intriguingly, however, the dose-dependent competition of the other FP assay was not impacted by incubation time, which is in great contrast with the FP assay reported here (Fig. 3*B*). This difference in the inhibition kinetics is intriguing and should be due to the drastically different ways that protein–protein interaction takes place in each case (22, 23). Given the dynamic feature of the inhibition for the FP assay reported here, it would be necessary to include, on every assay plate, positive and negative controls so that the effect of screened compounds can be evaluated safely and reliably.

We plan to use both this FP assay, which targets Nef’s activity on CD4, and the other FP assay, which targets Nef’s activity on MHC-I (27), in parallel to screen against the same compound libraries, which should help filter out false positives and allow us to identify true Nef inhibitors of potentially unique properties (*e.g.*, specific to one Nef function or dual functional).

## Experimental procedures

### Fusion protein design, expression, and purification

The α-Nef/σ2 fusion was constructed by fusing HIV-1 Nef (26–206, NL4.3) to the C terminus of α (1–398) subunit of AP2 *via* a flexible linker of 31 amino acids. Genes encoding the above α-Nef fusion (with a N-terminal 6xHis tag) and the σ2 subunit of AP2 were cloned into the two multiple cloning sites, respectively, of the pETDuet expression vector. *Escherichia coli* cells transformed with the plasmid were grown at 37 °C till *A*_600_ reached 0.8. Protein expression was then induced with 0.1 mM IPTG and continued at 16 °C overnight. Cells were then lysed using sonication. The protein of interest was purified sequentially through a nickel-nitrilotriacetic acid affinity column, a HiTrap Q anion exchange column, and finally a Superdex 200 size-exclusion column.

For MBP-CD4_CD_, the gene encoding 394 to 419 of CD4 was cloned into a pMAT9 vector. The MBP-CD4_CD_ protein was expressed overnight at 16 °C in NiCo21 (DE3) cells in terrific broth after induction with IPTG. Expressed protein was purified sequentially by a MBP affinity column, a HiTrap Q anion exchange column, and finally a Superdex 200 size-exclusion column.

For purifying the MBP protein, MBP-CD4_CD_, after being eluted from the MBP affinity column, was subjected to digestion by the Mpro protease, which cleaves at a site between MBP and CD4_CD_. The freed MBP was then purified similarly as MBP-CD4_CD_.

### FP assay

Purified α-Nef/σ2 was buffer exchanged into the assay buffer (50 mM Tris, 150 mM NaCl, 0.5 mM DTT, 0.01% Triton X-100, pH 8.0). A stock protein solution of 200 μM α-Nef/σ2 was then prepared and was subsequently used to create different dilutions. Assays were carried out in *Corning* 384-well black microplates (3820). In each well, 200 nM TMR-labeled CD4_CD_ peptide was mixed with α-Nef/σ2 at varied concentrations in a total volume of 15 μl. Incubation was done for 1 or 2 h at room temperature with minimal exposure to light. FP was then measured using the *EnVision* plate reader (*PerkinElmer*) with excitation at 535 nm and emission at 595 nm. Experiments were done in triplicates, and data was plotted using nonlinear regression as a function of protein concentration in a logarithmic scale using OriginLab (www.originlab.com).

The probes used were synthesized by *GenScript*, including: TMR-linear-CD4_CD_: TMR-MSQIKRLLSEKK; TMR-cyclic-CD4_CD_: cyclize-ASQIKRLLDEKKK(TMR)-cyclize; and TMR-mutant-CD4_CD_: MSQDKRDDSEKK.

For calculating the dissociation constant (K_D_), the noise, represented by the FP signal generated with TMR-mutant-CD4_CD_ being used as the probe, was first subtracted from the measured FP signal with TMR-cyclic-CD4_CD_. The adjusted data was then fitted to nonlinear regression for one-site binding using the following equation: Y = B_max_∗X/(K_D_ + X) (B_max_: maximum specific binding in the same units as Y).

### DMSO tolerance

Assay solutions were prepared containing 25 μM α-Nef/σ2, 200 nM TMR-cyclic-CD4_CD_, and different concentrations of DMSO (0–25%). After incubation at room temperature for 2 h, FP values were measured and recorded. All experiments were done in triplicates.

### Competition of TMR-cyclic-CD4_CD_ by unlabeled CD4_CD_ peptide or MBP-CD4_CD_

For competition using unlabeled CD4_CD_ peptide, a stock solution of 3 mM unlabeled CD4_CD_ peptide was first prepared. The stock solution was then serial-diluted (2-fold each) ten times. For making the final assay solutions, α-Nef/σ2 and TMR-cyclic-CD4_CD_ were first mixed and incubated at room temperature for 30 min. Then, unlabeled CD4_CD_ peptide was added (final concentrations: 25 μM α-Nef/σ2, 200 nM of TMR-cyclic-CD4, and varied concentrations of unlabeled CD4_CD_ peptide). The plate was incubated at room temperature for 2 h. FP values were recorded. All experiments were done in triplicates.

For competition using MBP-CD4_CD_ (or, in the control experiment, using MBP), an initial MBP-CD4_CD_ (or MBP) stock solution of 120 μM was prepared, and procedure used for measurements was similar as the above. The plate was read hourly for a total of 6 h.

### Determination of the *Z′* factor

Negative control contains 25 μM of α-Nef/σ2, 200 nM TMR-cyclic-CD4_CD_, and 2% DMSO. Positive control contains 25 μM α-Nef/σ2, 200 nM TMR-mutant-CD4_CD_, and 2% DMSO. Samples of positive controls and negative controls (66 wells each) were prepared in a 384-well plate and incubated at room temperature for 2 h. FP values were recorded using the plate reader. The *Z′* factor was calculated using the following equation:

$$Z^{\prime }=\;1\;-\;\frac{3\;\times \;\left(SD_{N}\;+\;SD_{P}\right)}{\left|\mu_{N}\;-\;\mu_{P}\right|}$$

where $\mu_{N}$ and $\mu_{P}$ are the averages of mP values of negative and positive controls, respectively. $SD_{N}$ and $SD_{P}$ are the standard deviations.

### Data analysis

All FP data were analyzed with nonlinear regression fitting using OriginLab. Statistical analysis was performed using ordinary one-way ANOVA in GraphPad Prism (www.graphpad.com). A *p* value of <0.05 is considered statistically significant.

## Data availability

All data generated or analyzed during this study are included in this published article.

## Conflict of interest

The authors declare that they have no conflicts of interest with the contents of this article.
